# Regulation of Obesity and Metabolic Complications by Gamma and Delta Tocotrienols

**DOI:** 10.3390/molecules21030344

**Published:** 2016-03-11

**Authors:** Lu Zhao, Xiefan Fang, Maurice R. Marshall, Soonkyu Chung

**Affiliations:** 1 Department of Food Science and Human Nutrition, University of Florida, Gainesville, FL 32611, USA; cauzhaolu@gmail.com (L.Z.); martym@ufl.edu (M.R.M.); 2 Department of Pediatrics, College of Medicine, University of Florida, Gainesville, FL 32611, USA; xiefanfang@ufl.edu; 3 Department of Nutrition and Health Sciences, University of Nebraska, Lincoln, NE 68583, USA

**Keywords:** obesity, tocotrienols, adipogenesis, energy sensing, apoptosis, inflammation

## Abstract

Tocotrienols (T3s) are a subclass of unsaturated vitamin E that have been extensively studied for their anti-proliferative, anti-oxidative and anti-inflammatory properties in numerous cancer studies. Recently, T3s have received increasing attention due to their previously unrecognized property to attenuate obesity and its associated metabolic complications. In this review, we comprehensively evaluated the recent published scientific literature about the influence of T3s on obesity, with a particular emphasis on the signaling pathways involved. T3s have been demonstrated in animal models or human subjects to reduce fat mass, body weight, plasma concentrations of free fatty acid, triglycerides and cholesterol, as well as to improve glucose and insulin tolerance. Their mechanisms of action in adipose tissue mainly include (1) modulation of fat cell adipogenesis and differentiation; (2) modulation of energy sensing; (3) induction of apoptosis in preadipocytes and (4) modulation of inflammation. Studies have also been conducted to investigate the effects of T3s on other targets, e.g., the immune system, liver, muscle, pancreas and bone. Since δT3 and γT3 are regarded as the most active isomers among T3s, their clinical relevance to reduce obesity should be investigated in human trials.

## 1. Introduction

According to the World Health Organization (WHO), obesity is defined as abnormal or excessive fat accumulation that may impair health. Body mass index (BMI), a person’s weight in kilograms divided by the square of his or her height in meters, is an inexpensive and easy-to-perform method of screening for weight category to classify normal (18.5–24.9 kg/m^2^), overweight (25.0–29.9 kg/m^2^) and obesity (30 kg/m^2^) in adults. BMI does not measure body fat directly, but research has shown that BMI is significantly correlated with direct measures of body fat [1,2]. Furthermore, BMI appears to be strongly correlated with various metabolic diseases associated with adiposity [3,4]. Obesity is now recognized as a disease and a serious public health issue worldwide. In the United States during 2011–2014, the prevalence of obesity was 17.0% in 2–19 year-olds and 36.5% in adults [5]. By using a simulation model, Wang *et al.* reported that the rising prevalence of obesity will affect 65 million more adults in the U.S. by 2030, and the total medical cost associated with the treatments of obesity and obesity-associated complications will increase by $48–66 billion/year, which is a heavy health and economic burden [6]. Obesity is categorized into two major types, hyperplastic (increase in adipocyte number) and hypertrophic (increase in adipocyte volume) obesity, both of which are regulated by genetic, endocrine, metabolic, neurological, pharmacological, environmental and nutritional factors [7]. Compared to other treatment strategies, such as drug intake and plastic surgery, dietary intervention is a safe and effective way to attenuate obesity and obesity-associated diseases [8]. In this review, we comprehensively update the emerging evidence that supports the use of tocotrienols (T3s) as a dietary supplement to attenuate obesity. We also thoroughly discuss the currently known molecular mechanisms that are modified by T3s in treating obesity. We propose that T3s, especially γ and δT3, are promising anti-obesity agents.

T3s are a subclass of vitamin E that possesses an unsaturated side chain, which can be further classified into four analogs (alpha, beta, delta and gamma T3 (αT3, βT3, δT3 and γT3)) based upon the substitution pattern of methyl groups on chromanol ring. In contrast to saturated vitamin E tocopherols (TPs), T3s only exist in a limited number of plants in nature. T3 are abundant and mainly present in palm oil (up to 800 mg/kg, mainly as αT3 and γT3), rice bran oil (mostly αT3 and γT3) and annatto oil (mostly of δT3) [9]. Annatto oil is unique and distinctive from palm or rice bran oil, because it contains only T3s, but little TPs. The ratios of T3s to TPs extracted from rice bran, palm and annatto sources are 50:50, 75:25 and 99.9:0.1, respectively [10]. Although T3s are being actively investigated for their preventive or therapeutic roles in some cancers, T3s have received less attention as dietary strategies to reduce obesity and its associated metabolic complications than their saturated counterpart TPs. Recently, however, research on T3s increased rapidly, reflecting the increasing interests in the potential health benefits of T3s using both pure and mixed T3 isomers. Notably, T3s have been documented to have potent anti-inflammatory and anti-cancer properties by modifying multiple molecular signaling pathways, which usually are not affected by TPs [10,11]. Moreover, T3s, especially γT3, lower the incidence of cardiovascular diseases, diabetes and cancer in animal models and human beings [12,13,14,15]. Emerging data indicate that T3s are promising anti-obesity agents. It has been reported that T3s reduce body weight [16] and improve plasma glucose and lipid hepato-protective effects in adults with nonalcoholic fatty liver disease [17], which is a complication associated with obesity. The pharmacological function and therapeutic potential of T3s to attenuate metabolic syndromes, such as cardiovascular disease, diabetes and lipid disorder, have been recognized and reviewed by several researchers [18,19]. However, little focus has been made on systemically reviewing the role of T3 for the prospective reversing of the on-set and development of obesity. On the other hand, despite several review articles showing that T3s exert anti-cancer effects on various cell types via modifying multiple signaling pathways, only scattered information is available regarding the mechanism of action of T3s in regulating obesity. Here, we comprehensively review the scientific literature regarding the impact of T3s on obesity, with a particular emphasis on the signaling pathways involved. We also focus on delta, gamma and mixed isomers of T3s rather than alpha and beta analogs based on stronger anti-obesity effects observed in γ and δT3.

## 2. Rationale for the Anti-Obesity Property of T3s

T3s have physically unique properties, and their potent anti-cancer and cholesterol-lowering effects are not exhibited by the saturated TP counterparts. T3s have been extensively investigated in the anti-cancer area due to their ability to selectively inhibit malignant tumor growth without harming normal cells via strong anti-proliferation [20,21,22,23,24,25,26], anti-oxidative [27,28,29,30,31,32] and anti-aging activities [33,34,35,36]. T3s also exert hypo-cholesterolemic and anti-sclerotic effects on humans, rats and mice (reviewed in [37]). Both *in vitro* and *in vivo* studies have shown that T3s reduce hepatic cholesterol production by inhibiting the activity of 3-hydroxy-3-methylglutaryl-CoA (HMG CoA) reductase, which is a key enzyme in the bio-synthesis of cholesterol. In addition, the T3-rich fraction (TRF) from palm oil decreases serum total cholesterol, LDL-cholesterol, apolipoprotein B and triglyceride levels in humans [38,39,40,41].

The fact that T3 isomers (γT3 and δT3) possess the abilities to inhibit hepatic cholesterol synthesis and cancer growth provides an exciting rationale that T3s would have a strong impact on reducing adiposity and fat cell formation. This is due to the fact that fat cell development (adipogenesis) shares many identical signaling pathways with tumor growth (tumorigenesis), including the Wnt, Stat, Akt, autophagy and mTOR signaling pathways [42]. In addition, changes of adipokines, a set of cytokines derived from adipose tissue, are strongly associated with a high risk of breast cancer, indicating that dysfunction of adipose tissue is tightly correlated with tumorigenesis [43,44]. An early study has found that γT3 downregulates the expression of adipogenic genes, *i.e.*, PPARγ and CEBPα, in breast cancer cells [45]. Taken together, the anti-cancer agent T3s, especially γT3, may reduce adipogenesis, which is a process highly resembling tumorigenesis.

## 3. Bioavailability and Accumulation of T3s

Compared to TPs, especially α-tocopherol (αTP), the bioavailability of T3s is relatively low. In healthy volunteers orally administrated with 300 mg mixed T3s (29% αT3, 55% γT3 and 14% δT3), their mean apparent elimination half-life (T_1/2_) values for αT3, γT3 and δT3 are 4.4, 4.3 and 2.3 h, respectively, which are 4.5–8.7-times shorter than that of αTP [46]. Similar to humans, the peak plasma concentration of γT3 is 2 μM in C57BL/6J mice receiving an oral administration of γT3 (90% purity) at 50 mg/kg. The plasma γT3 concentration drops dramatically to the baseline level in 4 h after orally giving 50 mg/kg γT3 [47]. In rats, the oral bioavailability of γT3 is as low as 9% [48]. The poor bioavailability of T3s is due to rapid metabolism in human liver microsomes [49]. The discrepancy in metabolism rate between T3s and TPs is due to higher affinity of αTP than T3s to α-tocopherol transfer protein (αTTP) in liver, resulting in preferential release of αTP to the blood stream and inhibited secretion of T3s [50]. Entrapment of non-αTP forms of vitamin E in hepatocytes leads to preferential hepatic metabolism of these molecules rather than αTP [51]. Recent studies have confirmed that pure T3 (either annatto T3s (90% δT3 and 10% γT3) [52] or a novel formulation of gamma-delta T3 (75% γT3 and 25% δT3) [53]) has superior bioavailability than TRF (mixed with α-tocopherol and other impurities) in human subjects due to the absence of αTP’s interference in absorption.

Despite low bioavailability, several studies have found that T3 slowly, but specifically accumulates in the peripheral tissues, including adipose tissue and skin [51,54]. When rats were fed with an AIN93G (a purified rodent diet) diet mixed with 0.2% T3s (2.5% αT3, 92% γT3 and 4.2% δT3) for 13 weeks, the level of γT3 ranged between 164 and 597 nmol/g in different types of fat pads [55]. Uchida *et al.* found that rats fed with a T3 mixture (33.9% αT3, 47.1% γT3 and 11% δT3) for six weeks followed by a vitamin E-depleted diet for one month, the αT3 and γT3 concentrations remained high in adipose tissue, indicating that αT3 and γT3 have slow degradation rates and that adipose tissue is a unique site to store T3s [56]. Dietary αTP is likely to counteract T3 accumulation in tissues, and it has been shown that 50 mg/kg αTP in the diet decreases αT3, but not γT3 accumulation in the adipose tissue of rats fed with a diet supplemented with 50 mg/kg αT3 or γT3 for eight weeks [56]. Accumulation of T3s in fat tissues indicates that the adipocytes may be the major site of action of T3s.

## 4. Effects of T3s on Obesity

### 4.1. Influences of T3s on Body Weight, Food Intake and Blood Profiles

Recently, several studies have directly or indirectly investigated the potential influences of T3s on obesity in laboratory animal models. Unlike the saturated forms of vitamin E, such as αTP, TRF at a daily dose of 10 mg for three weeks significantly reduced body weight in rats (Table 1) [57]. Our group has identified that 0.05% γT3 (90% purity) supplementation for four weeks significantly reduced high fat diet-induced body weight gain in young C57BL/6J mice [47]. Later on, Wong *et al.* found that δT3 (90% purity), but not αT3 (91.6% purity, <1% αTP) or γT3 (95% purity, <1% αTP), at a daily dose of 85 mg/kg for eight weeks significantly reduced abdominal adiposity and fat masses in diet-induced obese rats [58]. Through a body composition analysis, Ima-Nirwana *et al.* found that Sprague-Dawley (SD) rats that received γT3 at 60 mg/kg for eight weeks had decreased fat mass and increased bone calcium content [16]. Wistar rats fed a high fat diet with the administration of palm TRF (31.9% αT3, 24.8% γT3, 18.3% δT3) at 120 mg/kg/day for eight weeks had significantly decreased mesenteric fat mass by 20% without affecting epididymal and perirenal fat masses [59]. However, a 10%–20% loss of epididymal and perirenal fat was observed in obese male F334 mice fed with 10 mg rice bran T3 (RBT3) supplement (30% αT3 and 50% γT3) for three weeks [57]. These studies clearly demonstrated that T3s reduce body weight and decrease adipose tissue mass *in vivo*. Depending on the purity and isomers of T3s used in the dietary supplements, the effective dose of T3s to reduce body weight varies among studies [16,47,59,60,61]. In most studies, T3s had no significant impact on food intake [16,57,59]. Only one study found that administration of a TRF at 120 mg/kg for eight weeks increased food intake in rats [59]. In addition to the direct effects on body weight reduction, the anti-obesogenic effect of T3s is reflected in other parameters. Several *in vivo* studies have demonstrated that T3s reduce plasma concentrations of free fatty acids, triglycerides and cholesterol (Table 1) [59,60,62,63,64,65]. Furthermore, T3 supplement improves glucose and insulin tolerance (Table 1) [57,59,60,66].

### 4.2. Epidemiological Evidence; Observational and Clinical Trials

Although strong epidemiological evidence for a causal relationship between T3 intake and body weight reduction in humans is not yet available, many studies have shown that T3 intake is associated with decreased serum concentrations of total cholesterol and lipoproteins [12,67,68,69]. Qureshi *et al.* found that consuming 50 mg of a TRF (15% αT3, 35% γT3 and 25% δT3) for four weeks in hypercholesterolemic patients was inversely correlated with serum levels of total cholesterol and LDL, but did not alter serum levels of HDL and triglyceride [12]. In another study, hypercholesterolemic human subjects were given increasing doses of TRF from 25–200 mg/day in two 35-day treatment periods, and TRF at 100 mg/day caused the greatest declines in serum levels of total cholesterol, LDL and triglycerides, with reductions by 20, 25 and 12%, respectively [67]. However, in a recent placebo-controlled human trial using γT3 and δT3 at 120 mg/day for eight weeks, only the serum triglyceride level was lowered by 28%, and the total cholesterol, LDL and HDL levels remained unchanged between the treated and placebo groups [68]. The discrepancy in regulating lipid profiles by T3s among these studies may be attributed to the protocol employed, the purity of T3s, the length of the administration period, as well as the physiological conditions of the subjects.

Recently, several human studies reporting T3 effects on obesity-associated inflammation and insulin resistance have been published. Haghighat *et al.* have found that microalbuminuria and C-reactive protein (hs-CRP), which are indicators of acute inflammation, were reduced in patients with type 2 diabetes who received 200 mg/day T3 supplement (34.6% αT3 and 43.5% γT3) for eight weeks [70]. Similarly, Irandoost *et al.* have found beneficial effects of T3s on attenuating inflammation and insulin resistance in overweight or obese women [71]. In their study, 44 overweight or obese women were given a weight loss diet supplemented with either grape seed oil (with high levels of T3s) or sunflower oil (without T3s). Women given the grape seed oil supplement had lowered insulin resistance (HOMA-IR) scores and serum levels of hs-CRP and TNF-α compared to those given the sunflower oil supplement. In addition, a recent study conducted in 87 human subjects showed that a mixed T3 treatment (31% αT3, 56% γT3 and 13% δT3) at a dose of 400 mg/day for one year effectively reduced the hepatic echogenic response in patients with nonalcoholic fatty liver disease [17], which is a common complication of obesity. Taken together, these studies provide meaningful results to hypothesize the effects of T3s on improving the symptoms associated with obesity in humans. However, a mixture of T3s was used in these research works, which leads to a difficulty in identifying which isomer of T3s is the most beneficial. In addition, they failed to provide enough mechanistic support. Clinical studies using pure or mixed T3s are needed to further explore their roles in reducing obesity-associated complications.

## 5. Molecular Mechanisms of the Anti-Obesity Effects of T3s

Although many reports focusing on the molecular and cellular mechanisms responsible for the anti-tumor and anti-oxidative effects of T3s are available [10,11], little is known about the underlying mechanism of T3s involved in the regulation of obesity. Recently, several studies proposed the mechanisms involved in the anti-obesity effects of T3s, which were examined in different cell types and tissues. These mechanisms of action include (1) modulation of fat cell adipogenesis and differentiation; (2) modulation of energy sensing; (3) induction of apoptosis in preadipocytes and (4) modulation of inflammation. In addition to adipose tissues, several studies also explored the effects of T3s on immune system, liver, muscle, pancreas and bone.

### 5.1. Effect of T3s on Adipose Tissue

#### 5.1.1. Modulation of Fat Cell Adipogenesis

The influence of T3s on fat cell differentiation was first reported in 2009 when 3T3-L1 adipocytes were examined after being treated with T3s [72]. Uto-kondo *et al.* demonstrated that γT3, but not αT3 or αTP, reduced triglyceride content by 40% in 3T3-L1 adipocytes during their 21-day differentiation [72]. αT3 and γT3 significantly decreased insulin-induced mRNA expression of PPARγ, an important transcription factor for adipogenesis, by 55 and 90%, respectively, whereas αTP increased PPARγ expression [72]. This indicates that the effects of T3s on adipogenic differentiation is isomer specific, and a physiologically-relevant concentration of γT3 (2.4 μM) is able to inhibit the conversion of 3T3-L1 preadipocytes into adipocytes [72]. At a higher concentration (25 μM), treatments with T3s, especially γT3 and δT3, for eight days significantly reduced triglyceride accumulation and decreased the expression of adipogenic genes, *i.e.*, FAS and PPARγ, in 3T3-L1 cells [73]. Interestingly, γT3 and δT3 treatments in 3T3-L1 cells markedly increased mRNA and protein expression of CPT1 and UCP-2, but not HMG-CoA reductase, indicating that γT3 and δT3 may disrupt lipid metabolism during fat cell formation [73]. In addition to inhibiting adipogenesis, Wu *et al.* discovered that γT3 has the strongest anti-proliferative effect on 3T3-L1 cells and the lowest IC_50_ value (~13.91 μM) among all vitamin E analogs [74]. However, all of the studies described above used 3T3-L1 adipocytes as their cell model, which has three limitations. First, 3T3-L1 preadipocytes are mouse hybridoma cells; thus, it is difficult to distinguish the anti-adipocyte effect from the anti-tumor effect of T3s. Second, 3T3-L1 cells are committed preadipocytes that have lost their stem cell properties and are not good for differentiation studies. Finally, 3T3-L1 preadipocytes undergo post-confluent mitogenic expansion, which is not found in primary adipogenic precursor cells. Therefore, the effects of T3s on 3T3-L1 cells need further validation by using other *in vitro* and *in vivo* models.

Consistent with the results from 3T3-L1 cells, our research has identified the inhibitory effects of γT3 on adipogenesis by using primary human adipose tissue-derived stem cells (*h*ASCs), a physiologically more relevant adipocyte model to human than 3T3-L1 preadipocytes derived from rodents [75,76]. We also found that T3s inhibit adipogenesis in an isomer- and dose-dependent manner in differentiating *h*ASCs [77]. In particular, 1 μM of γT3 is able to significantly reduce triglyceride accumulation and decrease mRNA and protein expression of adipogenic marker genes, including PPARγ and aP2 [77]. Moreover, there was an increased expression of Pref-1 (a preadipocyte marker) in the adipose tissue and a decreased fat mass in γT3-fed obese mice, indicating that γT3 reduces fat mass via inhibition of preadipocyte differentiation [47]. Another study discovered that during early adipogenesis (0–48 h), γT3 treatment inhibits the mRNA expression of transcript factors CEBPα, but not CEBPβ [77], indicating that γT3 acts on a pathway downstream of C/EBPβ, but upstream of C/EBPα at the early stage of adipocyte differentiation. These observations support the finding that T3s decrease fat mass and body fat distribution *in vivo* (see Section 4.1). Notably, when fully-differentiated mature human adipocytes were treated with 5 μM of individual T3 isomers (αT3, γT3 or δT3), no noticeable dedifferentiation was found [77]. This indicates that T3s inhibit adipogenesis in adipose stem cells, but have no inhibition of lipogenesis in the pre-existing adipose tissue.

#### 5.1.2. Modulation of Energy Sensing

AMP kinase (AMPK) is an enzyme ubiquitously found in the body and has been characterized as a metabolic master switch that regulates the activities of a number of metabolism-controlling proteins (reviewed by Yun *et al.* [78]). Several phytochemicals, including quercetin, resveratrol and glabridin, inhibit adipogenesis via AMPK activation [79,80,81,82]. A recent study reported that T3s also activate AMPK [74]. In 3T3-L1 cells, γT3 at 20 μM increases phosphorylation of AMPK without changing the total AMPK level [74]. Similarly, our group has reported that a low concentration of γT3 (1 μM) is able to promote AMPK phosphorylation in differentiating *h*ASCs without altering the total level of AMPK [77]. In contrast, AMPK inhibitors, such as Compound **C**, significantly suppressed the γT3-induced ROS production and cell death in 3T3-L1 cells, indicating that the anti-adipogenic effect of γT3 on adipocytes may be mediated by AMPK signaling pathways. Furthermore, inhibition of AMPK by delivering AMPK-DN, a mutated form of AMPK, to cells significantly reversed the γT3-inhibited PPARγ activation and partially rescued the morphology of lipid-loaded adipocytes, which strongly indicates that activation of AMPK by γT3 suppresses adipogenic conversion [77].

As the downstream signaling cascades of AMPK, the mammalian target of rapamycin (mTOR) pathway has also been reported to be influenced by T3 treatments [83]. It has been suggested that fat cells sense the change of cellular energy status via the mTOR signaling pathway [84]. Interestingly, studies have found a positive correlation between T3s and mTOR in several cell lines [84,85]. For example, δT3 protects mouse and human hematopoietic progenitors from γ-irradiation through upregulation of mTOR and increases phosphorylation of its downstream effector 4EBP-1 [86]. In 3T3-L1 cells and neoplastic mammary epithelial cells, γT3 inhibits Akt phosphorylation, which is an upstream target of mTOR [72,87]. In *h*ASCs, we have demonstrated that γT3 decreases phosphorylation of mTOR P70S6 kinase, which is a downstream factor of mTOR, indicating that the anabolic pathways are downregulated by γT3 treatment [77]. However, another study from our group found that the insulin signaling cascades, *i.e.*, phosphorylation of IRS-1 and Akt, were markedly increased in the adipose tissue of γT3-fed mice and γT3-treated adipocyte compared to their respective controls [47], suggesting that γT3 exerts a different role in differentiated adipocytes by activating Akt instead of inhibiting Akt, as seen in preadipocytes.

#### 5.1.3. Induction of Apoptosis in Preadipocytes

Fat cell apoptosis is often concurrent with inhibition in adipogenesis [81,88]. It has been reported that T3s induce apoptosis in several types of cancer cells [89,90,91]. For example, Ahn *et al.* have reported that γT3 induces apoptotic cell death in cancer cells through downregulation of NFκB and its receptor-interacting proteins [92]. Recently, the apoptotic effect of T3s on preadipocytes has been revealed. Wu *et al.* have found that γT3 induces apoptosis in 3T3-L1 cells in a dose-dependent manner, which can be partially blocked by AMPK and capase-3 inhibitors [64]. Interestingly, a high dose of γT3 at 20 μM induces 3T3-L1 cell cycle arrest in S phase and decreases the number of cells in the G0/G1 phase [74]. Our group has found that after exposure to a low dose of γT3 at 1 μM for 10 days, differentiating *h*ASCs eventually underwent a capase-3-dependent apoptotic cell death [77]. In Wu’s and our experiments, a significant increase in the Bax/Bcl-2 ratio, an indicator of apoptosis, was observed, indicating that γT3-mediated alterations in Bax and Bcl-2 expression play an important role in promoting cell apoptosis [74,77]. In addition, we discovered that γT3 treatment in *h*ASCs increases cytosolic autophagosome (LC3II) accumulation and autophagic flux, indicating that γT3 promotes autophagy in differentiating adipocytes [77]. Collectively, γT3-mediated autophagy serves as a critical checkpoint that determines if preadipocytes become mature adipocytes or undergo an alternative apoptotic cell death.

#### 5.1.4. Modulation of Inflammation

Obesity is considered as low-grade inflammation in adipose tissue that leads to insulin resistance and other obesity-associated metabolic complications [93]. In the obese state, inflamed adipose tissue is often observed with abnormal productions of inflammatory cytokines and activation of inflammatory signaling in adipocytes [94]. It has been reported that injection of γT3 or δT3 at a dose of 10 μg/kg to BALB/c mice reduces lipopolysaccharide (LPS)-triggered inflammation [95]. Another study conducted by Tetsuro *et al.* found that 2.4 μM of γT3 significantly decreases the secretion of inflammatory cytokines, including MCP-1 and IL-6, in TNFα-treated 3T3-L1 adipocytes [96]. Additionally, γT3 treatment inhibits TNFα-mediated NFκB activation in adipocytes [97].

In young C57BL6/J mice, we found that γT3 supplementation is effective in reducing high fat diet-induced inflammation in adipose tissue by decreasing the expression of inflammatory genes, *i.e.*, MCP-1 [47]. In parallel, Justo *et al.* reported that a 5% rice bran extract supplement rich in T3s, when given to rats over 20 weeks, significantly reduces the expression of TNFα, IL-6, IL-1β and iNOS in visceral adipose tissue [98]. However, it is currently unclear how γT3 attenuates high fat diet-induced inflammation in adipose tissue. As reviewed by Jiang *et al.* [99], T3s, especially γT3, inhibit the activation of NFκB and STA6/3, as well as their downstream genes in various cell types, which may provide an explanation for how γT3 reduces inflammation in adipose tissue. This notion is supported by the study conducted by our group, which confirmed that activation of NFκB and MAP kinases by LPS is robustly attenuated in γT3-treated human adipocytes [47].

### 5.2. Effects of T3s on Immune System

In addition to directly reducing inflammation in adipose tissue, T3s inhibit inflammation by modulating the immune system. Recently, our group found that oral gavage of γT3 at 50 mg/kg for one week reduces LPS-induced secretion of several pro-inflammatory cytokines, *i.e.*, IL-6, TNFα and GM-CSF, in the plasma of C57BL/6J mice [47]. This is confirmed by two studies that found dietary supplementation with T3s, especially δT3, blocks LPS-induced TNF-α secretion in BALB/c mice and macrophages [95,100]. In addition, γT3 treatment partially reverses TNFα-mediated alteration in adiponectin secretion [96].

T cells play an essential role in immune responses, activation of which leads to an abnormal induction of cytokines [101]. T3s are potent compounds regulating T cell-mediated inflammation. Gu *et al.* have found that supplementation of 0.2% T3 mixture (13.1% αT3, 57.9% βT3 and 20.5% δT3) in diet for three weeks significantly increases the production of IgA and IgG in the lymphocytes harvested from spleen and mesenteric lymph node (MLN) regardless of the levels of concanavalin A (ConA) stimulation [102]. T3 feeding also decreases CD4^+^ T cells and the ratio of CD4^+^/CD8^+^ T cells in both spleen and MLN lymphocytes [102]. Moreover, 0.1% Tocomin (12.2% αT3, 2% βT3, 6.2% δT3 and 20.1% γT3) supplement for six weeks in four-month-old C57BL/6 mice increases the production of IL-1β in splenocytes [103]. At the molecular level, the effects of T3s on T cells are highly associated with multiple pathways that modulate cell proliferation and apoptosis [25,26]. Ren *et al.* have found that all T3 isomers enhance lymphocyte proliferation in aged mice with a potency ranking from αT3 > γT3 > δT3 [103]. Interestingly, γT3 and δT3 treatments suppress cell proliferation by arresting cell cycle in Jurkat T and CEM-SS cell lines derived from human T cells [25,60]. In addition, γT3 treatment inhibits ConA-induced activation of Swiss T cells by suppressing NF-κB and NF-κB-dependent gene expression [26]. Although γT3 is accumulated in lymphocytes, modulates intracellular glutathione and reduces oxygen concentration [26,104], the anti-inflammatory properties of γT3 are not related to its anti-oxidative effects [26]. Furthermore, γT3 treatment elevates mitochondrial ROS production, activates JNK, but inhibits phosphorylation of JNK and p-38 in Jurkat T cells. These changes lead to increased Bax and decreased Bcl levels, resulting in a caspase-dependent apoptosis in T cells [25]. However, transient exposure to γT3 for 4 h increases the survival and proliferation of T cells, which suggests that differential mechanisms of T3 in regulating human T cells may exist depending on the length of the treatment [26].

Obesity-related inflammation is associated with macrophage infiltration to adipose tissue [105]. T3s reduce inflammation in monocytes and inhibit differentiation of monocytes into macrophages via multiple pathways [104,106,107,108]. For example, αT3 significantly inhibits proteasome activity by increasing P27 and P57 protein expression in human monocytic cells (THP-1) [104]. Similarly, TRF treatment significantly reduces LPS-induced releases of NO and PGE_2_, as well as the excretion of proinflammatory cytokines by inhibiting NFκB activation in THP-1 cells [107]. Furthermore, αT3 and δT3 inhibit the excretion of adhesion molecules ICAM-1 and VCAM-1 from vascular endothelium cells, thus reducing monocyte cell adhesion [106,108]. In adipose tissue, T3s reduce inflammation via inhibition of macrophage infiltration [47]. Li *et al.* have found that TRF activates PPARs (*i.e*., PPARα and PPARγ) in murine macrophages, including Raw 264.7 and human THP-1 cells [38]. Wang *et al.* have shown that γT3 reduces LPS-stimulated IL-6 and GM-CSF secretions in bone marrow-derived macrophages by inhibiting CEBP/α and NFκB [109]. Our group has found that γT3 treatment reduces M1 macrophage polarization in bone marrow-derived macrophages, but has no impact on their differentiation into M2 macrophage [47]. Collectively, T3s reduce inflammation in adipose tissue, which is a possible mechanism contributing to their inhibition of obesity.

### 5.3. Effects of T3s on Liver

In addition to adipose tissue, liver is an important organ that regulates obesity by controlling lipid metabolism in the body. T3s play an important role in attenuating high fat diet-induced fatty liver [110]. It has been reported that a dietary supplement of γT3 for 16 weeks in rats effectively reduced high fat diet-induced hepatic triglyceride accumulation and decreased the size and number of fat vacuoles in the liver [59]. The same effects on liver were confirmed in other animals, including rabbits and chicken [110]. Additionally, γT3 supplement decreases high fat diet-induced inflammatory cell infiltration in the liver [111], leading to decreased obesity-induced inflammation.

In human HepG2 cells, Burdeos *et al.* have found that γT3 at 10–15 μM decreases mRNA and protein expression of fatty acid synthase, but increases mRNA expression of β-oxidation-associated genes, including CPT-1 and CYP3A4 [57]. Consistently, Muto *et al.* have shown that γT3 treatment in rat primary hepatocytes decreases the expression of hepatic steatosis-associated genes, including Chop and SREBP-1c, thus attenuating ER stress and reducing inflammation in the fatty liver [112]. Our group also observed a significant increase in the expression of genes associated with fatty acid oxidation in the livers of mice fed with high fat diet and γT3 supplement [47]. Li *et al.* have found that 0.2% TRF supplement in diet attenuates atherosclerosis in ApoE knockout mice through upregulation of LXRα and cholesterol transporters, such as ABCA1, in the liver [38]. Collectively, these data support the notion that γT3 downregulates biosynthesis of triglyceride and cholesterol in liver cells [113]. Because the majority of the orally-administrated T3s are metabolized in the liver [50], results from these studies indicate that γT3 supplement improves the obesity impaired liver function and whole body energy expenditure through increased hepatic fatty acid oxidation.

### 5.4. Effects of T3s on Other Tissues

T3s also exert their anti-obesity function on other tissues in addition to adipose tissue and liver [114]. T3s are well known for their neuro-protection function. Muscle is an important organ that utilizes glucose as the main energy source and regulates overall energy balance. Fang *et al.* reported that oral administration of 50 mg/kg TRF to Db/Db mice for two weeks increases the expression of β-oxidation-associated genes CPT2 and UCP3 by activating PPARδ in the muscle [15]. Similar to their effects on the liver, T3s increase energy expenditure in muscle, as well [114]. In addition, T3 supplement prevents pancreatic damage and restores obesity-impaired insulin secretion. Budin *et al.* have reported that feeding TRF at 200 mg/kg for 28 days in SD rats prevents fenitrothion-induced pancreatic damage through restoring the activities of anti-oxidative enzymes, including CAT and SOD [115]. Moreover, Kunnumakkara *et al.* have shown that γT3 administration inhibits pancreatic tumor growth by suppressing the NFκB-mediated inflammatory pathways in orthotopic nude mice [116]. The protective effects of γT3 on the pancreas may improve insulin production and reduce insulin resistance, which are complications in the obese state.

According to the most recent findings, excessive fat mass has a detrimental effect on bone health, such as increased risk in bone fracture [117,118,119], and T3s provide health benefits against both obesity and osteoporosis [47,77,120]. Adipogenesis and osteogenesis use mesenchymal stromal cells as their common precursors [121], and the balance of adipogenesis and osteogenesis is controlled by multiple pathways, including PPARγ and Wnt signaling. Thus, bone cell differentiation is decreased during fat cell formation [122,123]. Furthermore, a high fat diet induces inflammation, which promotes obesity and osteoclast [123,124]. In an obese state, adipokines, *i.e.*, leptin and adiponectin, decline in the systemic level, promoting osteoclast and bone resorption [123,124]. Similar to their beneficial effects on obesity, T3s effectively prevent osteoporosis and bone loss in several animal models [125,126,127]. It has been reported that TRF supplements are more efficient in preventing postmenopausal osteoporosis in estrogen-deficient rats compared to calcium fortification [125]. Moreover, a supplement of γT3 at 60 mg/kg for four months in healthy male rats markedly increases the volume, thickness, number and separation of trabeculae [128]. T3s promote bone health by multiple pathways. Nizar *et al.* have reported that low doses of γT3 (1 and 10 μM) prevent osteoblasts by reducing hydrogen peroxide-induced oxidative stress and apoptosis, leading to decreased osteoporosis [129]. Moreover, T3s prevent osteoclasts by reducing free radicals and inhibiting NFκB activation [120]. Ha *et al.* have found that αT3 suppresses the expression of RANKL, the NFκB receptor activator, in osteoclasts, and prevents RANKL-induced osteoclast differentiation through inhibiting c-fos expression [130]. More recently, Deng *et al.* have observed that the influence of γT3 on preventing ovariectomy-induced osteoporosis can be reversed by a daily administration of mevalonate, a HMG-CoA reductase inhibitor, in rats, indicating that γT3 prevents bone loss by inhibiting HMG-CoA reductase [131].

## 6. Limitations and Conclusions

The investigation of the effects of T3s on obesity and obesity-associated complications is a new and rapidly growing field. In the authors’ opinion, there are a few limitations in the existing literature regarding the anti-obesity effect of T3s, and additional study designs are needed to fill the following research gaps. First, many studies have used TRF for treatment instead of a single T3 isomer or mixtures with a known composition of each isomer. Thus, the pharmacological effects observed in these studies may not be caused by T3s alone, meaning that other components in TRF may produce a synergistic influence or antagonistic with T3s and affect the treatment outcomes. Furthermore, the different sources and compositions of TRF used in these studies make it difficult to directly compare results among them, and we are unable to identify the single most effective isomer of T3s. Future experiments are required to use individual T3 isomer for the determination of the exact mechanism of action of T3s. Second, as the majority of studies were performed *in vitro*, more *in vivo* studies in relevant animal models of metabolic syndrome or clinical human trials are needed to validate the anti-obesity effect of T3s. Third, most of the research interests are currently focused on the bioavailability of T3s and observational studies to describe the association between T3 intake and the outcome in reducing adiposity or improving blood lipoprotein profiles. Therefore, studies with mechanistic approaches that scrutinize the metabolic benefits and/or potential adverse effects are needed to characterize the legitimate anti-obesogenic effects of T3s.

**Figure 1:**
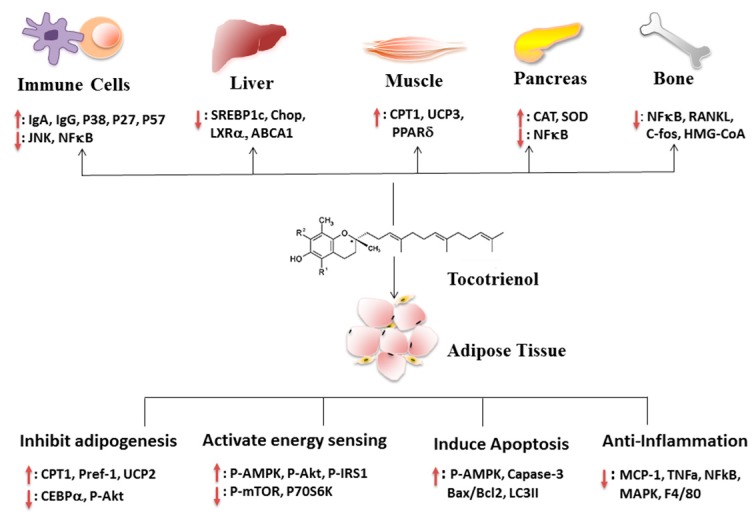
Mechanisms for the effect of tocotrienols (T3s) on obesity and obesity-associated complications. Briefly, T3s accumulate in adipose tissue and attenuate obesity mainly by: (1) inhibiting adipogenesis in adipose stem cells; (2) activating energy sensing in differentiating or maturate adipocytes; (3) enhancing apoptosis in preadipocytes; and (4) suppressing MAPK, NFκB and inflammatory cytokine secretion in inflamed adipocytes. In addition, T3s also exert their anti-obesity effects by reducing inflammation in immune cells, pancreas and bone, suppressing fatty acid and cholesterol synthesis in the liver and enhancing energy expenditure and antioxidant activity in muscle and pancreas.

There are continuous efforts to search for safe and effective dietary strategies that could intervene in obesity and its associated metabolic dysfunction. We and others have found that T3, especially γT3, improve obesity and obesity-associated complications by reducing adipogenesis and inflammation, as well as increasing apoptosis, energy sensing and energy expenditure (Figure 1). T3s modulate multiple signaling pathways mainly in adipose tissue and liver, with lesser effects on muscle and pancreas. Our latest study supports that γT3 inhibits the innate immune machinery and inflammation that produce potent cytotoxic cytokine IL-1β [132]. Given the fact that deregulation of autoimmune responses that are associated with inflammasome plays a key role in pathogenesis of multiple metabolic diseases, it provides a novel insight to control obesity and inflammation utilizing γT3. Although more studies are required to extrapolate the cellular and animal findings, there is a critical mass of studies supporting the notion that supplementation of γT3 or dietary γT3-enriched food would be a simple strategy to attenuate adiposity and to lessen obesity-mediated metabolic dysfunction, even without losing body weight.

## Figures and Tables

**Table 1:** Effects of tocotrienols (T3s) on body weight change, food intake, adipose tissue mass and serum lipid profile.

Parameter	Effects	Dose/Isomers/Duration	Models	Ref.
Body weight	―	120 mg/kg/TRF ^1^/8 weeks	Wistar Rat	[59]
	―	60 mg/kg/γT3 ^2^/8 weeks	SD Rat	[16]
	↓	10 mg RBT ^3^/3 weeks	F334 Rat	[57]
	↓	0.05% γT3 ^4^ in diet/4 weeks	C57BL/6J	[47]
	―	85 mg/kg/αT3 ^5^, or γT3 ^6^ or δT3 ^7^/8 weeks	Wistar Rat	[58]
Food intake	↑	120 mg/kg/TRF ^1^/8 weeks	Wistar Rat	[59]
	―	0.05% γT3 ^4^ in diet/4 weeks	C57BL/6J	[47]
	―	85 mg/kg/αT3 ^5^, or γT3 ^6^ or δT3 ^7^/8 weeks	Wistar Rat	[58]
Mesenteric fat	↓	120 mg/kg/TRF ^1^/8 weeks	Wistar Rat	[59]
	↓	10 mg RBT ^3^/3 weeks	F334 Rat	[57]
	↓	0.05% γT3 ^4^ in diet/4 weeks	C57BL/6J	[47]
Perirenal fat	―	120 mg/kg/TRF ^1^/8 weeks	Wistar Rat	[59]
Epididymal fat	―	120 mg/kg/TRF ^1^/8 weeks	Wistar Rat	[59]
	↓	10 mg RBT ^3^/3 weeks	F334 Rat	[57]
	↓	0.05% γT3 ^4^ in diet/4 weeks	C57BL/6J	[47]
	―	85 mg/kg/αT3 ^5^, or γT3 ^6^/8 weeks	Wistar Rat	[58]
	↓	85 mg/kg/δT3 ^7^/8 weeks	Wistar Rat	[58]
Body fat	↓	60 mg/kg/γT3 ^2^/8 weeks	SD Rats	[16]
	↓	120 mg/kg/TRF ^1^/8 weeks	Wistar Rat	[59]
	↓	5% rice bran extract ^8^/20 weeks	Zucker rice	[61]
	―	85 mg/kg/αT3 ^5^, or γT3 ^6^/8 weeks	Wistar Rat	[58]
	↓	85 mg/kg/ δT3 ^7^/8 weeks	Wistar Rat	[58]
Serum or plasma total cholesterol	―	120 mg/kg/TRF ^1^/8 weeks	Wistar Rat	[59]
	↓	20 μM/kg/γT3 ^9^/4 weeks	New Zealand rabbit	[62]
	↓	0.09% γT3 ^10^ in diet/5 weeks	Wistar Rat	[63]
	↓	50 μg/g TRF ^11^ in diet/6 weeks	Wistar Rat; Cholesterolemic Pigs	[64,65]
	―	85 mg/kg/αT3 ^5^, or γT3 ^6^/8 weeks	Wistar Rat	[58]
	↓	85 mg/kg/δT3 ^7^/8 weeks	Wistar Rat	[58]
Fasting plasma glucose	↓	120 mg/kg/TRF ^1^/8 weeks	Wistar Rat	[59]
	↓	200 mg/kg BW/TRF ^12^/8 weeks	SD Rats	[66]
	―	85 mg/kg/αT3 ^5^, or γT3 ^6^/8 weeks	Wistar Rat	[58]
	↓	85 mg/kg/δT3^7^/8 weeks	Wistar Rat	[58]
Plasma free fatty acid	↓	120 mg/kg/TRF ^1^/8 weeks	Wistar Rat	[59]
	↓	0.09% γT3 ^9^ in diet/5 weeks	Wistar Rat	[63]
	―	85 mg/kg/αT3 ^5^	Wistar Rat	[58]
	↓	85 mg/kg/γT3 ^6^ or δT3 ^7^/8 weeks	Wistar Rat	[58]
Plasma triglyceride	↓	120 mg/kg/TRF ^1^/8 weeks	Wistar Rat	[59]
	↓	10 mg RBT ^3^/3 weeks	F334 Rat	[57]
	―	85 mg/kg/αT3 ^5^, or γT3 ^6^/8 weeks	Wistar Rat	[58]
	↓	85 mg/kg/δT3 ^7^/8 weeks	Wistar Rat	[58]

Note: ―: no changes; ↓: decrease; ↑: increase; ^1^ TRF: tocotrienol rich fraction (31.9% αT3, 24.8% γT3, 18.3% δT3); ^2^ γT3: gamma tocotrienol (purity unavailable); ^3^ RBT3: rice bran tocotrienol (30% αT3, 50% γT3, 6% tocopherols (TPs)); ^4^ γT3: gamma tocotrienol (purity 90%); ^5^ αT3: alpha tocotrienol (91.6% purity, <1% αTP); ^6^ γT3: gamma tocotrienol (95% purity, <1% αTP); ^7^ δT3: delta tocotrienol (90% purity); ^8^ rice bran extract: (99 mg/kg TPs and 174 mg/kg T3s; composition unavailable); ^9^ γT3: gamma tocotrienol (purity unavailable); ^10^ γT3: gamma tocotrienol (>88% purity); ^11^ TRF: tocotrienol rich fraction extracted from palm oil (10%–20% αTP, 15%–20% αT3, 30%–35% γT3 and 20%–25% δT3); ^12^ TRF: palm oil tocotrienol rich fraction (mostly T3s, small portion of TPs; composition unavailable). Unless otherwise stated, the information about the TP contents in the above isomers is not available.
